# Adjustments in the motor unit discharge behavior following neuromuscular electrical stimulation compared to voluntary contractions

**DOI:** 10.3389/fphys.2023.1212453

**Published:** 2023-06-01

**Authors:** Riccardo Borzuola, Stefano Nuccio, Martina Scalia, Martina Parrella, Alessandro Del Vecchio, Ilenia Bazzucchi, Francesco Felici, Andrea Macaluso

**Affiliations:** ^1^ Department of Movement, Human, and Health Sciences, University of Rome “Foro Italico”, Rome, Italy; ^2^ Department Artificial Intelligence in Biomedical Engineering, Faculty of Engineering, Zentralinstitut für Medizintechnik (ZIMT), Friedrich-Alexander University Erlangen-Nürnberg, Erlangen, Germany

**Keywords:** HDsEMG, NMES, motor unit (MU), electrophysiology, MU recruitment, motor unit discharge rate

## Abstract

**Introduction:** The application of neuromuscular electrical stimulation superimposed on voluntary muscle contractions (NMES+) has demonstrated a considerable potential to enhance or restore muscle function in both healthy and individuals with neurological or orthopedic disorders. Improvements in muscle strength and power have been commonly associated with specific neural adaptations. In this study, we investigated changes in the discharge characteristics of the tibialis anterior motor units, following three acute exercises consisting of NMES+, passive NMES and voluntary isometric contractions alone.

**Methods:** Seventeen young participants participated in the study. High-density surface electromyography was used to record myoelectric activity in the tibialis anterior muscle during trapezoidal force trajectories involving isometric contractions of ankle dorsi flexors with target forces set at 35, 50% and 70% of maximal voluntary isometric contraction (MVIC). From decomposition of the electromyographic signal, motor unit discharge rate, recruitment and derecruitment thresholds were extracted and the input-output gain of the motoneuron pool was estimated.

**Results:** Global discharge rate increased following the isometric condition compared to baseline at 35% MVIC while it increased after all experimental conditions at 50% MVIC target force. Interestingly, at 70% MVIC target force, only NMES + led to greater discharge rate compared to baseline. Recruitment threshold decreased after the isometric condition, although only at 50% MVIC. Input-output gain of the motoneurons of the tibialis anterior muscle was unaltered after the experimental conditions.

**Discussion:** These results indicated that acute exercise involving NMES + induces an increase in motor unit discharge rate, particularly when higher forces are required. This reflects an enhanced neural drive to the muscle and might be strongly related to the distinctive motor fiber recruitment characterizing NMES+.

## 1 Introduction

In recent years, exercise protocols based on neuromuscular electrical stimulation (NMES) have provided a considerable number of functional and physiological benefits in both healthy and individuals with neurological or orthopedic disorders (Maffiuletti, 2010). Most commonly, studies have reported an increase in skeletal muscle strength following training protocols involving NMES, with the greatest effectiveness obtained by delivering NMES while voluntarily performing movement exercises (NMES+), as described in two recent reviews (Paillard, 2018; Borzuola et al., 2022). Interventions based on NMES + have shown the potential to improve muscle force generating capacity even to a greater extent than NMES only or voluntary exercise training alone (Paillard et al., 2005; Herrero et al., 2010; Bezerra et al., 2011; Labanca et al., 2018; Labanca et al., 2022; Dörmann et al., 2019). It has been argued that increases in force production capacities, besides being accompanied by changes in muscle morphology, could primarily be attributable to neural adaptations (Moritani and DeVries, 1979; Enoka, 1988; Folland and Williams, 2007).

In the last 2 decades, several attempts have been made to identify the sites of the neural adaptations induced by NMES training. Since the early studies, major attention has been given to the alterations occurring in the spinal circuitry following acute and chronic exercise (Aagaard et al., 2002; Scaglioni et al., 2002). Two neurophysiological studies indicated that the effectiveness of NMES + intervention could be related to an increase in spinal excitability resulting from plastic changes in Ia reflex pathways (Lagerquist et al., 2012; Borzuola et al., 2020). Specifically, an acute potentiation of the Hoffmann (H) reflex responses in the soleus muscle was reported following voluntary isometric ankle plantar flexions paired with superimposed NMES applied on the posterior tibial nerve (Lagerquist et al., 2012), and on triceps surae motor points (Borzuola et al., 2020).

At a supraspinal level, inconsistent findings have emerged when assessing cortical adaptation to NMES using transcranial magnetic stimulation (TMS). Some authors indicated an increased excitability of the primary motor and premotor cortices after an acute intervention based on NMES alone or superimposed on voluntary contractions (Khaslavskaia and Sinkjaer, 2005; Barsi et al., 2008; Schabrun et al., 2012), while another study reported no changes in corticospinal excitability following an acute bout of NMES superimposed on voluntary contractions of the ankle plantar flexors (Lagerquist et al., 2012). Such discrepancies have been frequent in studies involving TMS, particularly when assessing corticospinal adaptation following volitional training (Weier et al., 2012; Christie and Kamen, 2014; Coombs et al., 2016; Giboin et al., 2018). Nonetheless, both the assessment of spinal excitability, and corticospinal activation, involve the evaluation of evoked responses (H-reflexes and motor evoked potentials) and do not allow to capture changes in volitional neural activity. For this reason, some authors argued that assessments performed using these techniques might provide only a partial portrait of the neural adaptations induced by exercise (Škarabot et al., 2021). Furthermore, there is still little if any knowledge of the changes at the motor unit level following exercise protocols, including NMES interventions (Duchateau and Enoka, 2011).

Recent advances in electrophysiology have devised a non-invasive method to assess the activity of large populations of motor units (MUs) during voluntary muscle contractions. This is achieved by accurate decomposition of high-density surface electromyographic (HDsEMG) recordings which involves the application of multichannel grids of evenly spaced electrodes over the muscles of interest (Holobar and Zazula, 2007; Merletti et al., 2008; Del Vecchio et al., 2020). HDsEMG provides a reliable measure of MUs discharge times which, in turn, are used to estimate several MUs characteristics such as discharge rate, recruitment-derecruitment threshold and input-output gain of the motoneuron pool. A recent study, involving HDsEMG measured on the tibialis anterior muscle, indicated that strength enhancements following short-term resistance training was accompanied by increased MUs discharge rate and decreased MUs recruitment threshold (Del Vecchio et al., 2019). These findings are in agreement with those of a previous work which investigated the discharge rate of motor units of the vastus medialis and lateralis muscles, by means of intramuscular EMG, following voluntary strength training (Vila-Chã et al., 2010). Conversely, the study of Lecce et al. reported that an acute exercise involving whole-body vibration induced no changes in MU discharge properties (Lecce et al., 2023). However, to the best of our knowledge, there is no study investigating adaptations occurring at the motor unit level following a NMES intervention.

Therefore, the aim of this study was to investigate the acute changes of the tibialis anterior muscle MUs behavior following NMES superimposed on voluntary isometric ankle dorsi flexions (NMES + ISO) compared to passive electrical stimulation only (NMES) and voluntary sub-maximal isometric dorsi flexions only (ISO). Previous studies indicated that MU recruitment pattern during NMES is random and NMES can activate high threshold MUs (Gregory and Bickel, 2005; Bickel et al., 2011), at various force levels, while voluntary contractions follow the physiological “size principle” for MU recruitment, with low threshold MUs being recruited first (Henneman et al., 1965). Based on this evidence, as well as the reported neural adaptations following NMES exercises, we hypothesized that MUs discharge rates would be greater following passive NMES and NMES + ISO at higher force levels in which both low and high threshold MUs are involved. Conversely, we expected higher discharge rates at lower force levels, in which low threshold MUs are mainly contributing to the contraction, following ISO. We also hypothesized that increases in motor unit discharge rates would be accompanied by a decrease in MU recruitment threshold. Finally, based on the increase in spinal motoneuron excitability reported in previous studies following superimposed electrical stimulation (Lagerquist et al., 2012; Borzuola et al., 2020), we expected that the input-output relationship of the spinal motoneuron would be increased following the NMES + ISO condition. Altogether, these findings would provide a portrait of the adjustments occurring at the motor unit level following different type of acute exercise.

## 2 Methods

### 2.1 Participants

Seventeen young healthy volunteers (eleven males and six females, mean ± SD age: 28 ± 5 years, mass: 69 ± 11 kg, height: 1.74 ± 0.07 m), with no history of neurological or orthopaedic disorders, participated in the study. A statistical power analysis was performed *a priori* with a software (G*Power v. 3.1.9.4) to determine the sample size (*α* = 0.05, power = 0.95, effect size = 0.4) as indicated by Cohen. (1992) based on previous studies analyzing neurophysiological adaptations in response to similar acute exercise protocols (Lagerquist et al., 2012; Borzuola et al., 2020; Lecce et al., 2023). The participants did not have any experience with NMES exercise before performing the experimental session and were asked to not engage in any strenuous activity in the 24 h before the experimental session. The study was approved by the University of Rome “Foro Italico” ethics review board (CAR 86/2021) and all participants gave informed written consent before participating.

### 2.2 Experimental design

**FIGURE 1:**
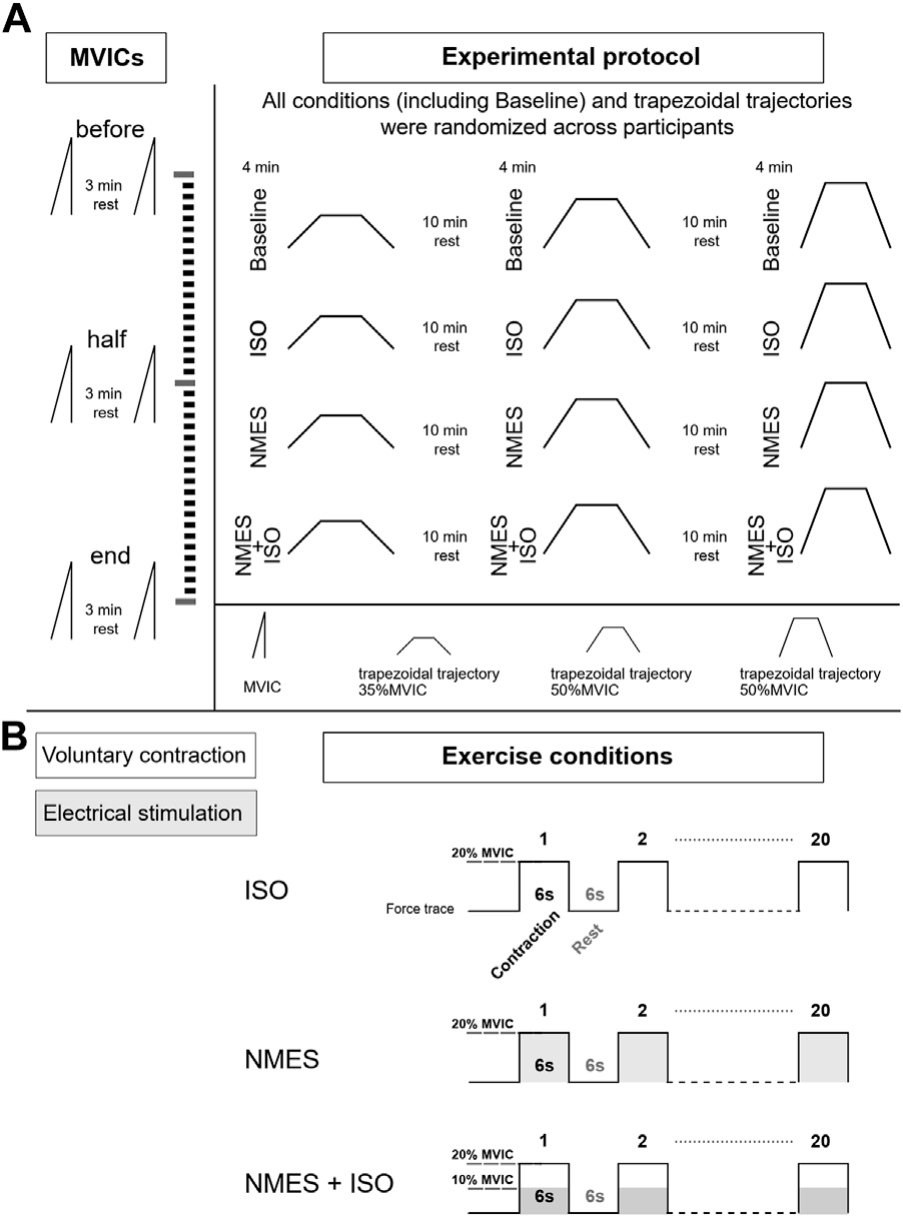
Experimental design. **(A)** Diagram of the experimental protocol. During the experimental session, participants performed three MVICs (before at half and at the end of the experimental conditions) while exercise conditions (Baseline, ISO, NMES, NMES + ISO) were repeated three times and immediately followed by one of the three trapezoidal force trajectory (35, 50, 70% MVIC). **(B)** Description of the exercise conditions. Each condition involved 20 intermittent isometric contractions (6 s contraction/6 s rest) at 20% MVIC as illustrated by example of the force traces.

The experimental session included three experimental conditions: a) NMES applied on the tibialis anterior muscle (NMES) b) NMES superimposed on voluntary isometric contraction of the ankle dorsi flexor muscles (NMES + ISO) and c) voluntary isometric contraction of the ankle dorsi flexor muscles only (ISO). Moreover, a Baseline condition, during which participants did not perform any exercise, was carried out throughout the experiment. Participants performed each condition, including Baseline, in a random order during one experimental session which lasted around 3 h. Each exercise condition involved 20 intermittent contractions at 20% of maximal voluntary isometric contraction (MVIC) (6 s contraction/6 s rest) for a total duration of 4 min. During Baseline, participants remained at rest in a seated position for 4 min. Participants were allowed a recovery period of 10 min between all conditions. The number and duration of contractions used in this protocol was selected to prevent development of muscle fatigue (Gueugneau et al., 2017; Grosprêtre et al., 2018), while at the same time inducing neural adjustments as demonstrated in previous literature (Lagerquist et al., 2012; Borzuola et al., 2020; Borzuola et al., 2023). All the procedures were performed on participants’ dominant leg as described by Botter et al. (2011). Leg dominance was determined as the limb preferred for hopping or kicking a ball (Holmbäck et al., 2003). The experimental session involved the simultaneous assessment of ankle dorsi flexors forces and tibialis anterior muscle myoelectrical activity with high-density surface EMG (HDsEMG), while participants performed submaximal trapezoidal ankle dorsi flexions at three different force targets (35, 50, 70% MVIC). Participants were allowed a period of familiarization with the trapezoidal tasks for a total duration of approximately 20 min in which they performed at least three attempts for each force target and a low divergence of the force trajectory from the given trapezoidal template was exhibited. Each condition was followed by one trapezoidal contraction at a specific target force which was determined in random order. Hence, the three experimental conditions, as well as the Baseline assessments, were repeated three times, once for each trapezoidal trajectory. MVIC assessments were repeated at three different times (before, at half and at the end of the experimental conditions) to check if any fatigue had arisen throughout the protocol. The experimental design is illustrated in Figure 1.

### 2.3 Force recordings

During the experiment, participants seated comfortably on a chair which was secured to an adjustable ankle dynamometer (OT Bioelettronica, Turin, Italy). The knees were flexed at 90° (0° = full knee extension), the hip at 90° (0° = neutral hip position), and the ankle at 0° of ankle plantar-dorsi flexion (0° = foot orthogonal to the shank axis). The ankle and the foot of the dominant leg were firmly secured, with padded Velcro straps, to the adjustable foot plate of the ankle dynamometer while the contralateral leg rested on a footrest positioned next to the dynamometer. The foot plate was connected with a calibrated force transducer (CCT Transducer, Turin, Italy), located perpendicularly below the footplate. The analogue signal recorded by the force transducer was amplified with a 500 gain, sampled at 2000 Hz, and converted to digital data by a 16-bit external analogue-to-digital converter (Sessantaquattro, OT Bioelettronica, Turin, Italy). Force and HDsEMG signals were acquired with the software OTbiolab (OT Bioelettronica, Turin, Italy). After a period of warm-up and familiarization during which participants performed 15–20 submaximal isometric contractions (about 50% of perceived maximal contraction), the MVIC assessment involved increasing the force of the dorsi flexor muscles gradually from zero to a maximum over 3 s and maintaining the maximal value for ∼3 s before relaxing. Participants were verbally encouraged to promote their maximal effort. Two MVIC attempts were performed, with each attempt being separated by 3 min rest intervals to minimize the effect of fatigue. MVIC was chosen as the largest 500 ms average achieved within a force recording. Assessment of MVIC was then used to define a target isometric plantarflexion force as 20% MVIC, which was used for the conditioning contractions, and the force target of the trapezoidal trajectories. The trapezoidal contractions consisted of a linear increase in force at 5% MVIC s−1 (recruitment phase), followed by 10 s of constant force at relative target force (plateau phase) and finally a linear decrease in force at 5% MVIC s−1 (derecruitment phase). Participants were required to exert force with their ankle dorsi flexor muscles and accurately match the trapezoidal force path displayed on a monitor placed 1 m from their eye-level. A real-time visual feedback of both the force output and the expected trajectory were provided to participants at a constant visual gain with the software OTbiolab.

### 2.4 NMES

**FIGURE 2:**
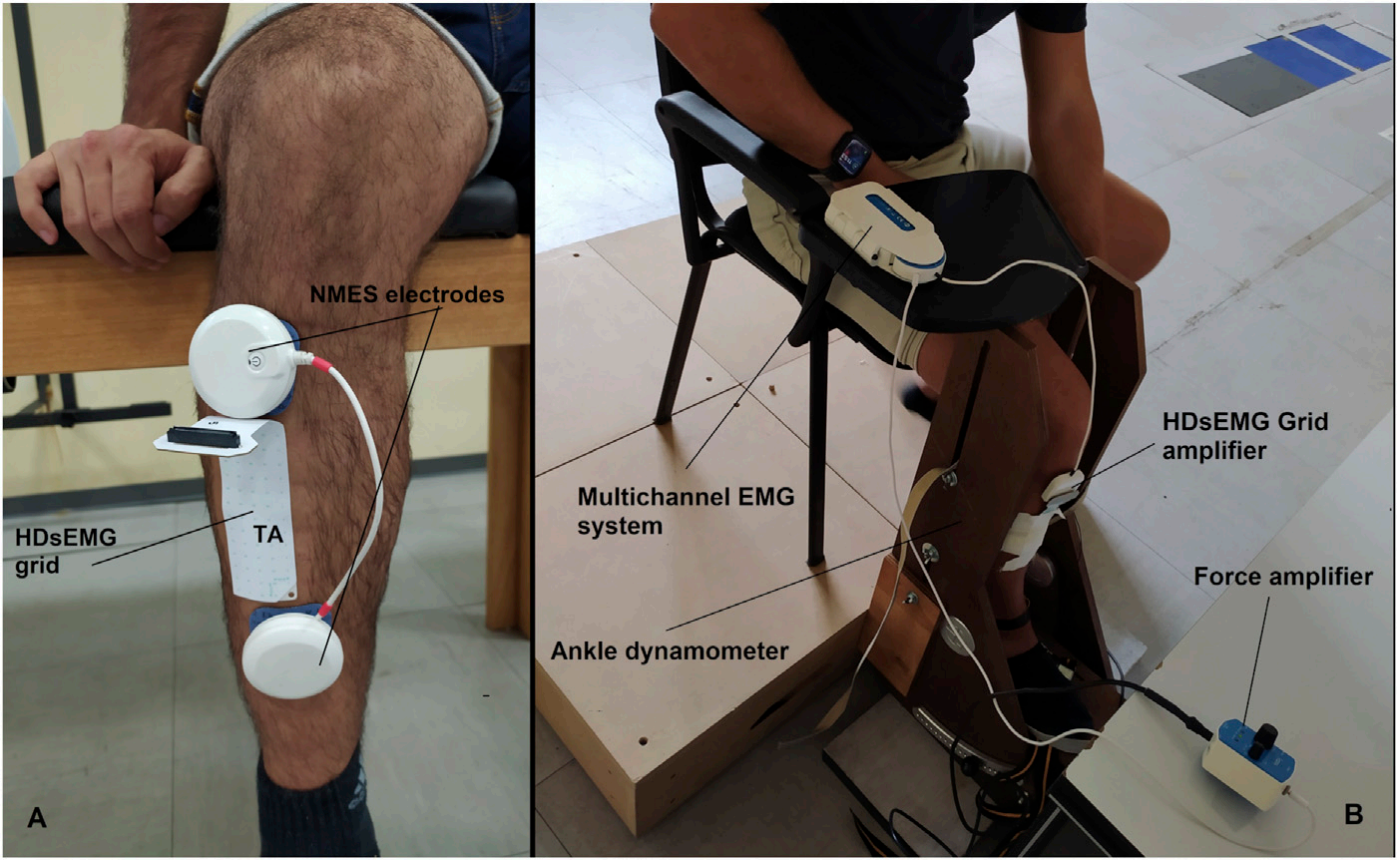
**(A)** Electrodes and HDsEMG grid placement on the tibialis anterior muscle. **(B)** Experimental setup.

A portable muscle stimulator (Chattanooga Wireless Professional, DJO Global, Vista, CA, United States) was used to deliver NMES over the tibialis anterior muscle. NMES was administered either passively or superimposed on voluntary contraction of the dorsi flexor muscle. The stimulator produced a rectangular, balanced biphasic pulse and was constantly handled and controlled by the operator. Two self-adhesive electrodes (Compex Dura-Stick plus, 50 × 50 mm, DJO Global, Vista, CA, United States) were used to deliver the stimulation. The anode was placed over the motor point of the tibialis muscle, whereas the cathode was placed distally on the same muscle as illustrated in Figure 2. The motor point of the tibialis anterior muscle was identified at the beginning of the experimental session with a hand-held anode ball electrode in accordance with the electrical stimulator user’s guide. NMES was delivered with a pulse frequency of 50 Hz and a pulse width of 400 μs per phase to generate higher forces while promoting the highest comfort during electrical stimulation as reported in previous investigation (Baldwin et al., 2006). The current pulse intensity of the stimulation was manually adjusted in accordance with each participant’s tolerance. Before the beginning of the experimental conditions, participants familiarized with the electrical stimuli for about 10 min at low intensity. During NMES, current pulse intensity was increased (average NMES intensity: 19.7 mA; range 10.1–31.7 mA) until the passively stimulated ankle dorsi flexion reached the target force at 20% MVIC. During ISO, participants were required to match the target force of 20% MVIC by voluntarily contracting their dorsi flexor muscles. In the NMES + ISO condition, current pulse intensity was set to generate half of the target force (10% MVIC; average NMES intensity: 14.7 mA; range 8.5–26.6 mA) and participants were asked to voluntarily contract their dorsi flexor muscles to achieve the full target of 20% MVIC. During NMES + ISO, participants were asked to relax their tibialis anterior muscle before the first and after the tenth contraction while the investigator adjusted the pulse intensity to ensure that the force produced corresponded to half of the target force throughout the entire NMES + ISO condition.

### 2.5 HDsEMG signal recording

Surface EMG signals were recorded in monopolar derivation from the tibialis anterior muscle using a high-density adhesive grid of 13 × 5 equally spaced electrodes [gold-coated, 1 mm diameter, 8 mm inter-electrode distance (IED); OT Bioelettronica, Turin, Italy]. An operator identified the perimeter of the muscle by manual palpation and positioned the grid over the most innervated areas of the distal area of the tibialis anterior muscle (Figure 2), as described in previous studies (Del Vecchio et al., 2019; Casolo et al., 2020). Participants’ skin over the selected area of the muscle was shaved, lightly abraded, and cleansed with 65% ethanol to ensure the best conductivity of the HDsEMG signal. The grid was secured over the skin of tibialis anterior muscle parallelly to the lateral margin of the tibia using a disposable bi-adhesive foam layer which was provided with holes adapted to the HDsEMG grid (SpesMedica, Battipaglia, Italy). The foam layer holes were filled with conductive paste (SpesMedica) to ensure skin-electrode contact. The reference electrode of the grid was positioned on the medial malleolus of the tested leg with an ankle strap moisten for the purpose. The recording grid was connected to a multichannel amplifier (Sessantaquattro, OT Bioelettronica, Turin, Italy). The HDsEMG signals detected were sampled at 2000 Hz, band-pass filtered (3 dB bandwidth, 10–500 Hz) and converted to digital data by the multichannel amplifier.

### 2.6 Force analysis

The analogue force signal was converted to newtons (N) and low-pass filtered with a cut-off frequency of 15 Hz (4th order, zero-lag, Butterworth) during offline analysis. We applied a gravity correction to account for the signal offset. The trapezoidal contractions which showed clear pre-activations (≤0.5 N from the baseline force signal in the 150 ms prior to the force onset) or countermovement were excluded from further analysis (Del Vecchio et al., 2019; Casolo et al., 2020; Nuccio et al., 2021).

### 2.7 HDsEMG signal processing

**FIGURE 3:**
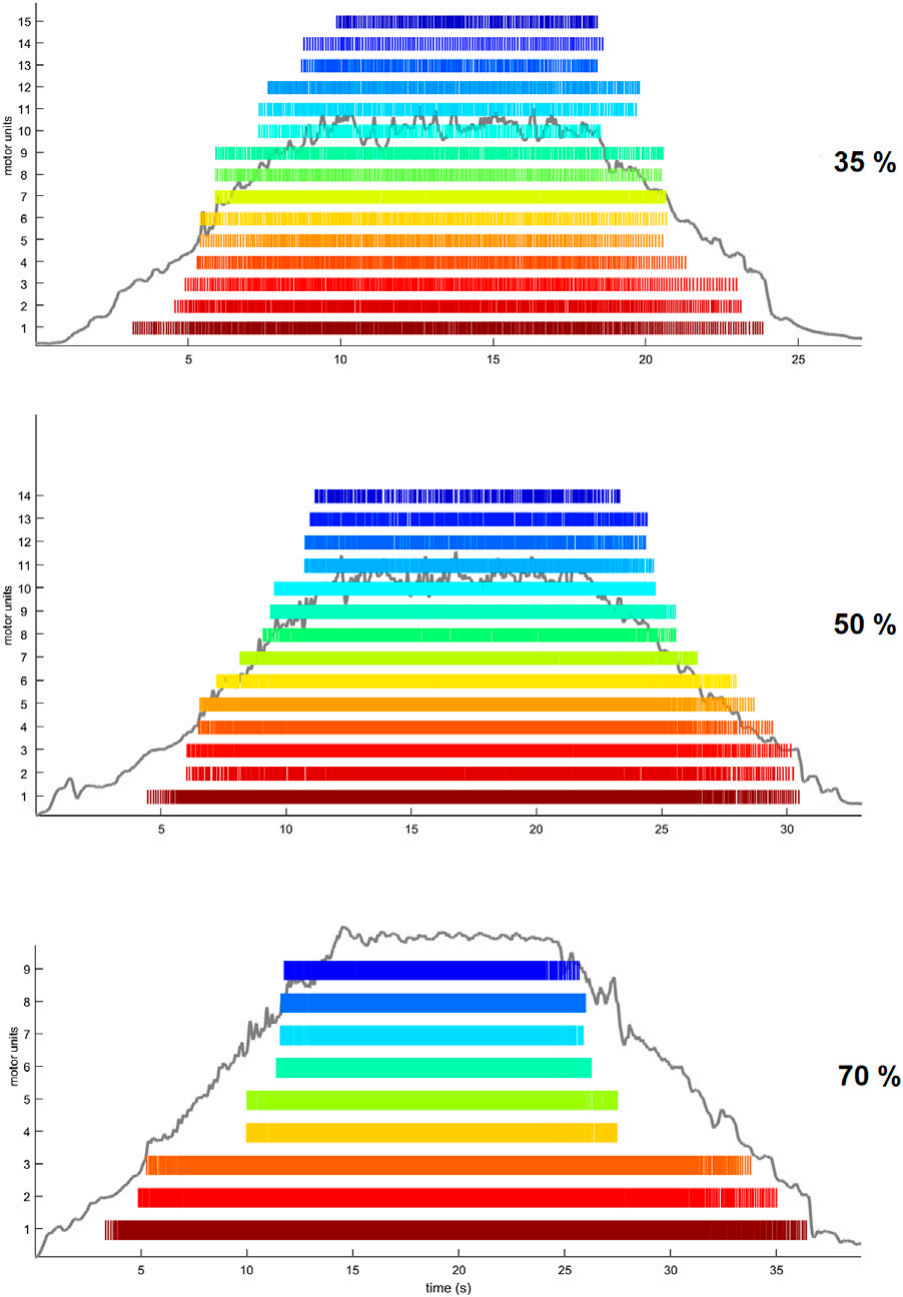
Examples of trapezoidal force trajectories at 35%, 50% and 70% MVIC. Raster plots are displaying the spike trains of MUs identified by the BSS decomposition analysis from one participant.

We performed the analysis of HDsEMG signals recorded from the grid located on the distal portion of the TA muscle. After being low-pass filtered offline (20–500 Hz, Butterworth, 2nd order) a validated convolutive blind source separation (BSS) technique (Holobar and Farina, 2014) was used to decompose the HDsEMG signal into individual motor unit discharge times. A decomposition accuracy of 95% was used during the BSS procedure. Motor unit discharge times were then converted into binary spike trains which were manually inspected by the investigator to remove those showing poor signal quality (Holobar and Zazula, 2007). In particular, MUs showing a pulse-to-noise ratio (PNR) ≤30 dB or an inter-spike time interval higher than 2 s were excluded from further analysis (Holobar and Farina, 2014). Moreover, duplicates of MUs were deleted within each trial. HDsEMG signal decomposition and manual editing was performed with the DEMUSE tool software (v4.9; The University of Maribor, Slovenia) developed on Matlab (v2020a, Mathworks Inc., Natick, MA, United States). In Figure 3, we show raster plots indicating the spike trains of identified MUs during the three different trapezoidal contractions. A MU tracking procedure was used to improve the robustness of the comparisons between the conditions. MUs were tracked across all conditions (Baseline, ISO, NMES, NMES + ISO), separately for each trapezoidal force target, using the approach described in Martinez-Valdes et al. (2017). Briefly, motor unit tracking was based on a correlation analysis of the motor unit action potential, which was extracted by spike-triggered averaging from the HDsEMG signals at the discharge times of the motor units identified by blind-source separation (Holobar and Zazula, 2007). The motor unit action potentials with high correlation values (>0.7) were visually inspected. MU discharge characteristics were extracted only from tracked MUs that were identified in all four conditions. MU recruitment threshold (RT) and derecruitment threshold (DERT) were determined as the relative force levels (% MVIC) at which a single MU discharged its first and last action potential, respectively. We calculated average motor unit discharge rate (DR, pulses per second, pps) for recruitment (DRREC), plateau (DRPLAT) and derecruitment (DRDER) phases and globally for entire trapezoidal contraction. DRREC and DRDER were calculated from the first four and the last four discharge times, respectively. DRPLAT was computed using the discharge times identified during the whole plateau phase.

The regression slopes for the increase in MUDR from recruitment to target torque (mean DR minus DRREC, ∆DR_R-T_) relative to the increase in torque from the recruitment threshold (target torque (30, 50, 70% MVIC) minus RT, ∆F_R-T_), were calculated as an indirect estimate of the input–output gain of the tibialis anterior muscle motoneuron pool (Martinez-Valdes et al., 2018; Boccia et al., 2019). This analysis provides indirect information on the synaptic input received by the motoneuron pool and represents the sum of all the inputs converging from the different levels of the nervous system (Martinez-Valdes et al., 2018; Del Vecchio et al., 2019). Changes in the ∆DR_R-T_—∆F_R-T_ torque relationship indicates a modification in the gain of motoneurons when force generation is required (Martinez-Valdes et al., 2020). All identified MUs, including those which were not tracked across conditions, were used to compute the input-output gain (Nuccio et al., 2021).

### 2.8 Statistical analysis

Statistical analysis was performed using IBM SPSS 24.0 (IBM Corp., Armonk, NY, United States) and Jamovi 2.2.5 (The Jamovi project, Sydney, Australia). The Shapiro-Wilk test was used to assess the normality of distribution of the reported variables. When variables did not show a normal distribution, they were log-transformed to meet the assumption of normality before applying the statistical test. To detect differences in DR patterns, MUs RT and DERT of tracked MUs, separate linear mixed models (GAMLj pack: General Analyses for the Linear Model in Jamovi) were used for each target forces (35, 50, 70% MVIC) as a function of condition (Baseline, ISO, NMES, NMES + ISO) with a fixed effect intercept and a participant specific random intercept was applied using restricted maximum likelihood estimation. The analysis was performed using only tracked MUs which were identified in all four conditions. Significance of the fixed effect was assessed by an F-test using Satterthwhaite’s approximation for the degrees of freedom. A Bonferroni-Holm correction was applied when needed to account for multiple comparisons. A linear regression analysis was performed to model the relationship between ∆DR_R-T_ and ∆F_R-T_. This analysis was performed to obtain an estimate of the participants input-output gain of the tibialis anterior muscle motoneuron pool. Participant-specific regression slopes were compared between conditions using a repeated measure ANOVA. Paired t-tests were used to compare the number of identified MUs across conditions and between different trapezoidal target forces. A repeated measure ANOVA was used to compare the MVIC assessments at three different times (before, at half and at the end of the experimental conditions). For all statistical tests, significance level was set to 0.05. Data are reported as means ± SD unless stated elsewhere.

## 3 Results

### 3.1 MUs decomposition

**FIGURE 4:**
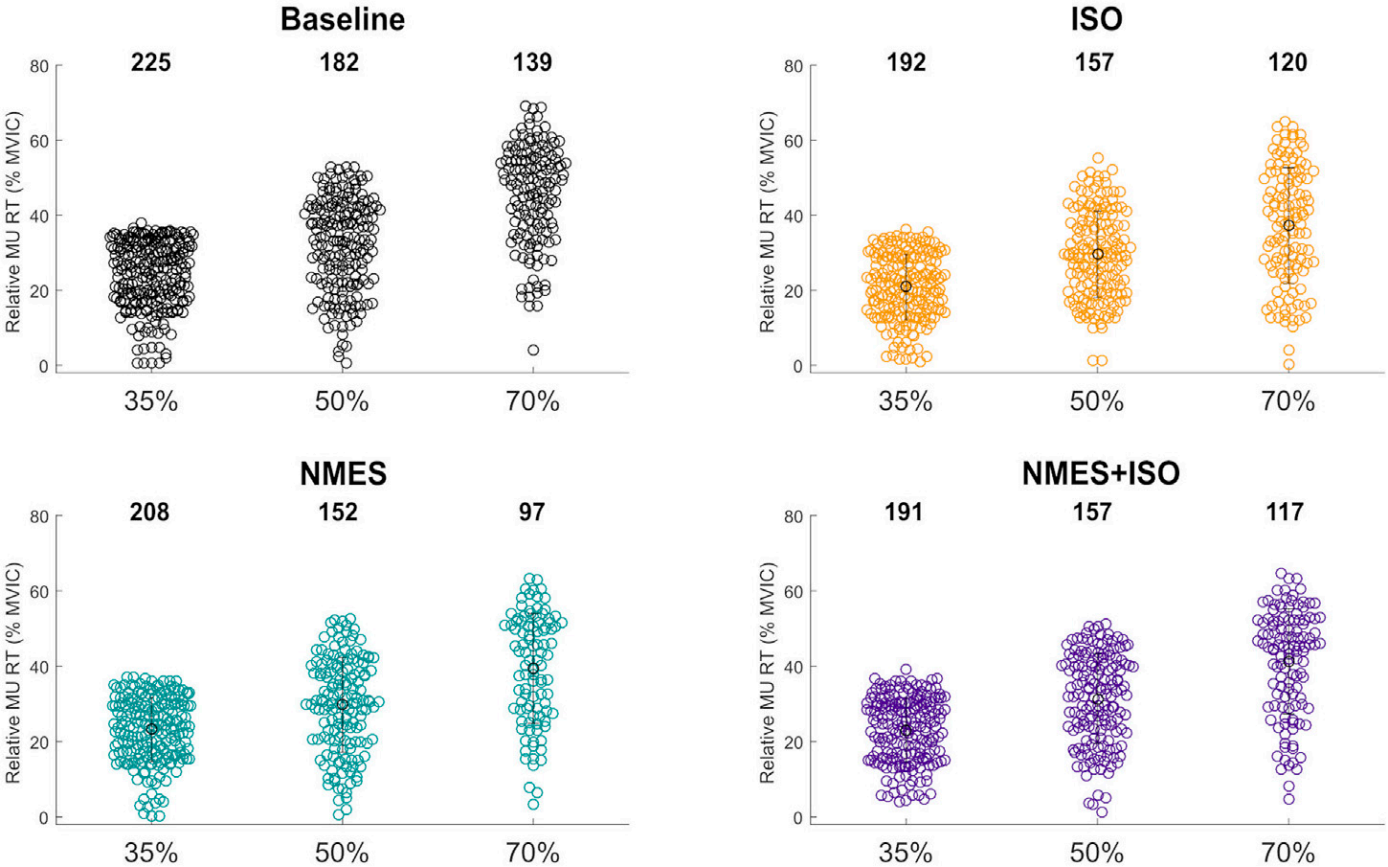
Number and distribution tibialis anterior identified MUs. Swarm plots representing the totality of MUs identified in the tibialis muscle in all conditions and for each trapezoidal target force. The distribution of the identified MUs is expressed according to the relative MU RT.

A total of 1937 MUs were identified in the TA of the participants. The distribution and number of MUs for each condition and trapezoidal contraction level are shown according to their relative RT in Figure 4. Of all MUs, 776 MUs (approximately 40%) were tracked across all four conditions (Baseline, ISO, NMES, NMES + ISO) at the three target forces. The average number of identified MUs was significantly different between trapezoidal target forces (*p* = 0.001). Specifically, the average number of MUs identified during the trapezoidal contractions at 35% MVIC were higher than the MUs identified at 50% MVIC (*p* = 0.032) and 70% MVIC (*p* = 0.001) target force. Additionally, the average number of MUs identified at 50% was significantly greater than the MUs identified at 70% (*p* = 0.003).

### 3.2 MUs properties

**FIGURE 5:**
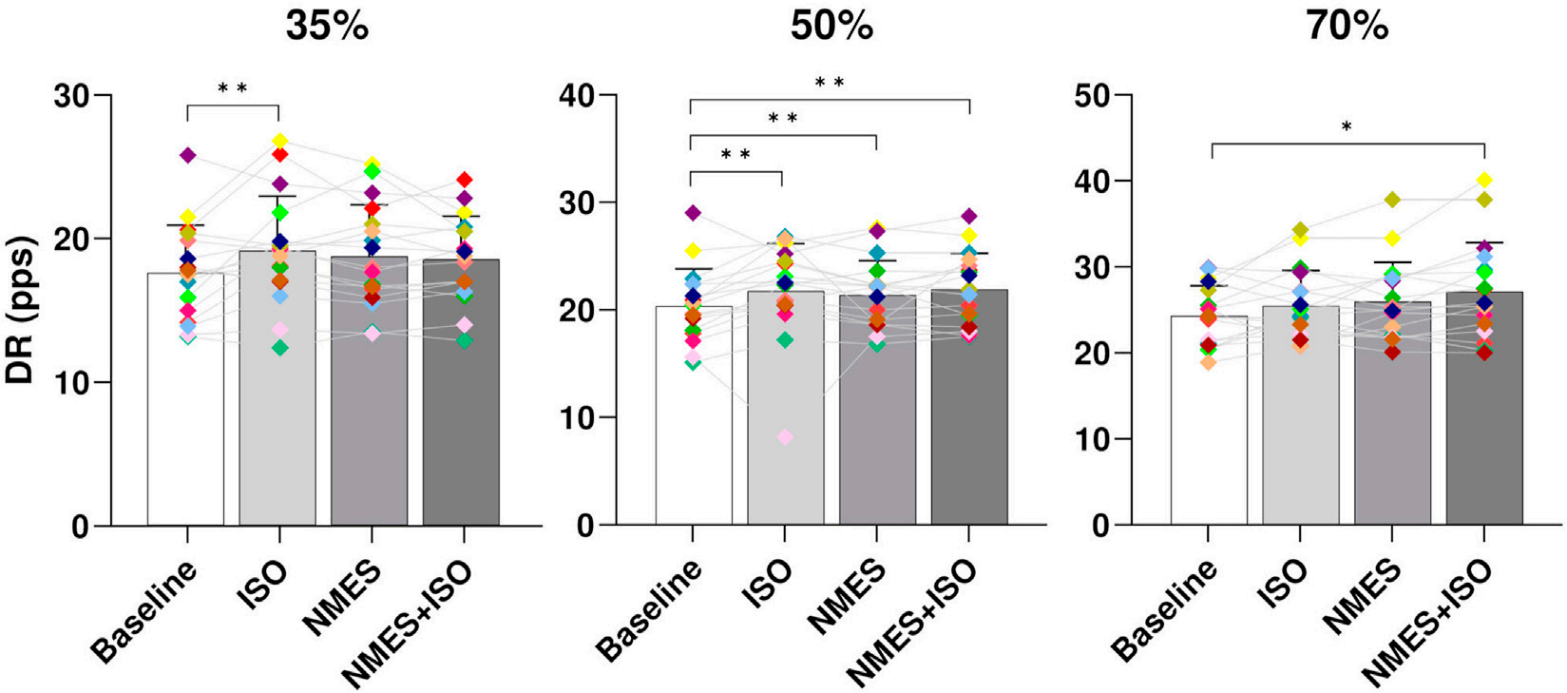
Differences in global MU DR. Bar plots showing the average global MU DR (averaged across the entire trapezoidal contraction) for condition (Baseline; ISO; NMES; NMES + ISO) and for trapezoidal target force (35%; 50%; 70% MVIC). Participant-specific values are displayed as diamonds of different colours; *p < 0.05, **p < 0.01.

MU DR. At all target forces there was a significant effect of condition on global DR (35% MVIC, *p* < 0.001; 50% MVIC, *p* < 0.001; 70%, *p* < 0.003). Post-hoc analysis showed significant differences between Baseline (17.9 ± 3.3 pps) and ISO (19.2 ± 3.8 pps, *p* < 0.001) at 35% MVIC. At 50% MVIC there was a significant increase of global DR in all experimental conditions (ISO, 22.4 ± 4.4 pps, *p* < 0.001; NMES, 21.7 ± 3.2 pps, *p* = 0.001; NMES + ISO, 21.9 ± 3.4 pps, *p* < 0.001) compared to Baseline (20.3 ± 3.5 pps). At 70% MVIC, a significant increase of DR was found only in the NMES + ISO (26.8 ± 3.7 pps, *p* = 0.03) compared to Baseline (25.4 ± 2.8 pps). Data are presented in Figure 5.

When considering DR during the different phases of the contraction there was a significant effect of condition during the plateau phase (DRPLAT) and only at 35% and 50% MVIC (35% MVIC, *p* = 0.009; 50% MVIC, *p* < 0.001). The *post hoc* tests reported a significant increase of DRPLAT after the ISO condition (20.4 ± 3.8 pps, *p* = 0.005) compared to Baseline (19.2 ± 3.5 pps) at 35% MVIC. At 50% MVIC, DRPLAT was significantly greater following all the experimental conditions (ISO, 24.1 ± 5.4 pps, *p* < 0.001; NMES, 23.3 ± 3.7 pps, *p* = 0.019; NMES + ISO, 23.7 ± 3.8 pps, *p* = 0.002) compared to Baseline (21.8 ± 3.9 pps). The statistical analysis exhibited no significant effect of condition for DRREC (35% MVIC, *p* = 0.618; 50% MVIC, *p* = 0.431; 70%, *p* = 0.741) and DRDEREC (35% MVIC, *p* = 0.084; 50% MVIC, *p* = 0.936; 70%, *p* = 0.432).

### 3.3 MU RT and DERT

**FIGURE 6:**
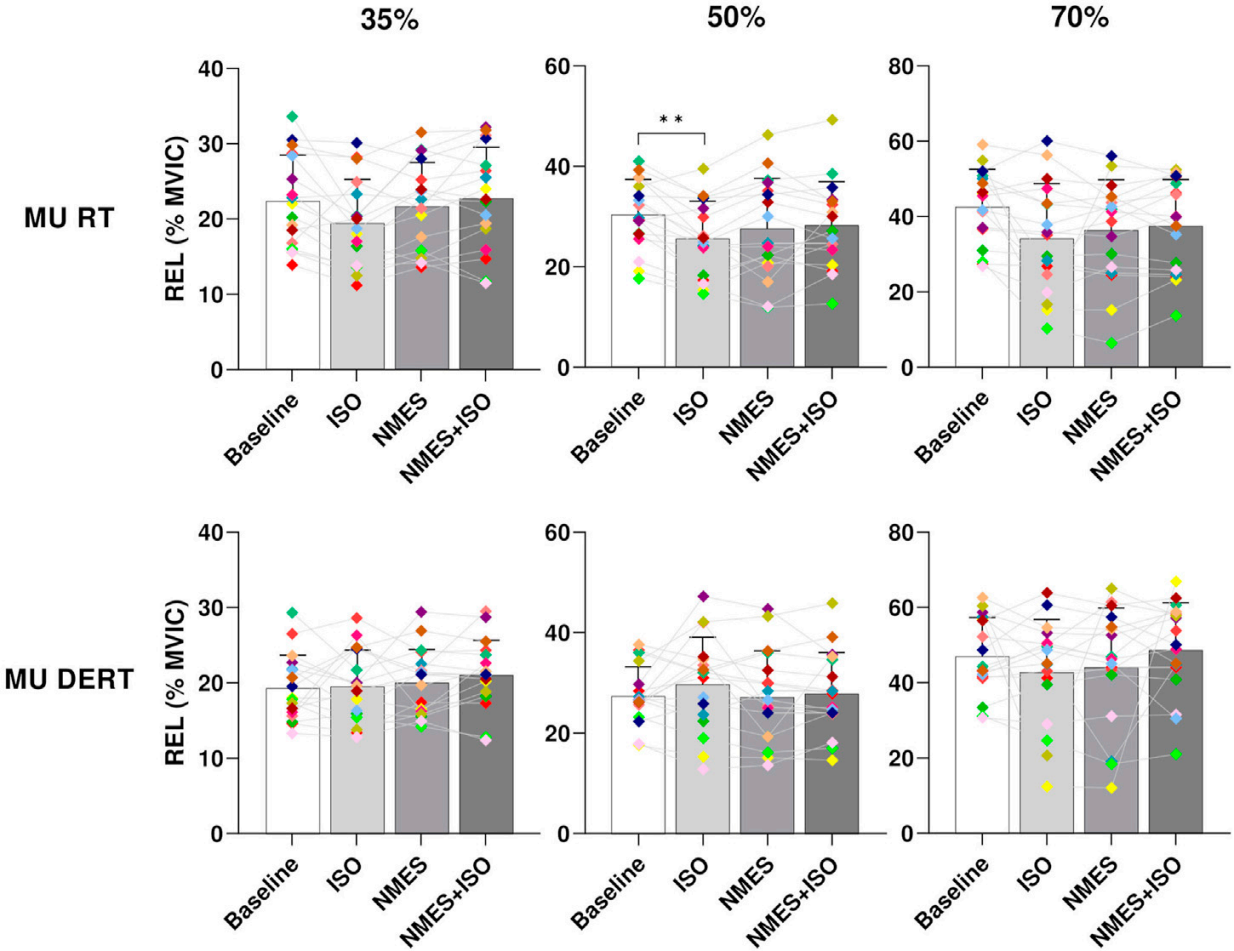
Differences in MU RT and DERT. Bar plots showing the average MU RT and DERT of tibialis anterior for each condition (Baseline; ISO; NMES; NMES + ISO) and for each trapezoidal target force (35%; 50%; 70% MVIC). Participant-specific values are displayed as diamonds of different colours; *p < 0.05, **p < 0.01.

Results on MU recruitment and derecruitment threshold are presented in Figure 6. There was a significant effect of condition on relative MU recruitment threshold (RTREL) at 35% and 50% MVIC (35% MVIC, *p* = 0.031; 50% MVIC, *p* = 0.006). However, the *post hoc* analysis revealed differences only at 50% MVIC indicating a RTREL significantly decreased after ISO (25.5 ± 7.4, *p* < 0.001) compared to Baseline (30.8% ± 6.9% MVIC).

### 3.4 Input-output gain of the TA motoneuron pool

**FIGURE 7:**
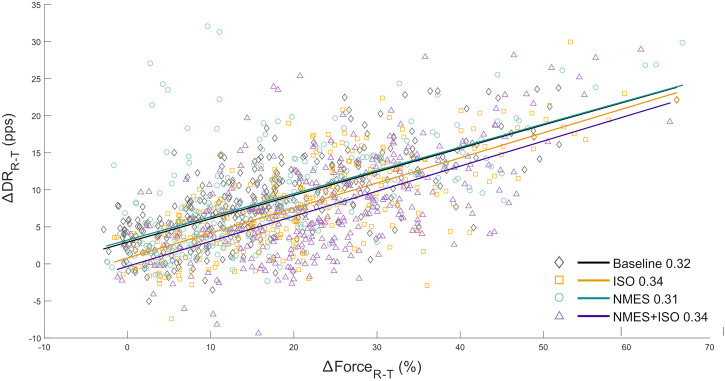
Input-output gain of the tibialis anterior motoneuron pool. a) scatter plots showing the change in ankle dorsi-flexor muscles force (∆F_R-T_ from recruitment to target) as a function of the change in MU DR (∆DR_R-T_ from recruitment to target) of the totality of MUs identified in the tibialis anterior. ∆F_R-T_ is expressed as values relative to MVIC. Regression lines for each experimental condition and relative slopes (rate of change of MU DR as a function of the rate of change of force) are reported.

The relationship between the variation in discharge rate (∆DR_R-T_) with respect to the change in voluntary force (∆F_R-T_) provided an estimation of the synaptic input converging to the TA motoneurons. ∆DR_R-T_ and ∆F_R-T_ were linearly correlated in all participants, The repeated measures ANOVA indicated that there was no main effect of condition (*F* = 0.66, ηp2 = 0.06, *p* = 0.53) on the slopes of the regression lines which represents the rate of change in MU DR as a function of the rate of change in force. The regression lines are reported in Figure 7.

### 3.5 MVIC

The repeated measure ANOVA did not reveal any main effect of time (*F* = 0.57; ηp2 = 0.05; *p* = 0.61) on MVIC values, suggesting that no fatigues had arisen throughout the experimental protocol. MVIC measurements are reported in Table 1.

**TABLE 1:** MVIC measured at three times throughout the experimental protocol. Data are presented as means ± SD.

	Before	Half	End
MVIC (N)	221 ± 79	222 ± 82	219 ± 77

## 4 Discussion

The findings of this study indicate that three different exercise modalities (ISO, NMES and NMES + ISO) induced specific neural adjustments in the discharge behavior of tibialis anterior motor units. Specifically, global values of MU DR were greater at 50% of trapezoidal target forces following the three experimental conditions compared to baseline values. This result was observed also when evaluating DR during the plateau phase of the trapezoidal contraction. At 35% target force, only the ISO condition produced a significant increase in MU DR and DRPLAT. Interestingly, at the highest trapezoidal target force of 70% MVIC, the NMES + ISO led to an increase in global DR compared to baseline, whereas the other two conditions did not produce any significant change in DR. This finding indicates that at higher target forces NMES + ISO might have the potential to generate specific adaptations at the MU level which, in turn, could represent the basis for functional improvements related to NMES + ISO exposure. A possible contributor to the increase in DR at higher forces might be represented by the distinctive motor fiber recruitment patterns that has been commonly hypothesized when analyzing NMES (Jubeau et al., 2007; Bickel et al., 2011). The motor unit adjustments reported at high and moderate force levels appear to be in line with the hypothesis that NMES + ISO contractions may involve high threshold MUs as well as low threshold MUs. This seems to be confirmed by the fact that ISO produced a higher DR at low force levels in which a greater number of low threshold MUs contribute to the contraction. However, contrary to our hypothesis, we found no difference in MU DR at high force levels following passive NMES which can activate high threshold MUs in addition to low threshold MUs in order to generate the same force level. This could be related to the superficial localization of low-threshold MUs in the TA (Lexell et al., 1983) as NMES activate predominantly muscle fibers with axonal branches in proximity to the stimulating electrodes (Vanderthommen et al., 2000). As the level of force that was reached in each trapezoidal trajectory did not differ across conditions, it could be argued that increase in MU DR after acute exercises might be related to muscle fatigue (Macaluso et al., 2000), assuming that active MUs would require to compensate with greater DR to achieve equivalent force outputs. However, several authors reported that in presence of fatigue, particularly following submaximal tasks (including 20% MVIC), the mean discharge rate of MUs significantly decreased (McManus et al., 2015; Hwang et al., 2020). It has been suggested that in presence of fatigue, earlier-recruited MUs are selectively inhibited and decrease their DR, while the concurrent increase in the excitation to the motoneuron pool leads to the recruitment of new MUs to sustain the force output (Enoka et al., 1989; Stewart et al., 2011; McManus et al., 2015). Furthermore, the acute protocol of the present study has been shown not to induce any neuromuscular fatigue (Gueugneau et al., 2017; Borzuola et al., 2020), as it could have constituted a potential bias for the physiological assessments. Therefore, modulation of MU DR following exercise could be related to both intrinsic properties of the motoneurons as well as excitatory/inhibitory inputs received by the motoneurons including afferent feedbacks and descending drive (Enoka and Duchateau, 2017). Several authors have indicated that monoamine-facilitated persistent inward currents (PICs) are likely involved in the modulation of MU DR given their strong influence on motor neuron responsiveness to ionotropic inputs, hence, motor neuron excitability (Heckman and Enoka, 2012). This has been recently reported during voluntary exercise (Orssatto et al., 2021) or in response to local vibration (Lapole et al., 2023), although further investigation is required to consolidate the interpretation on the underlying mechanisms. In addition, some researchers have indicated that changes in the portion of common input (PCI), which is an estimate of the relative proportion between the common inputs converging to the motoneuron pool and the total synaptic inputs at low frequencies, as well as MU synchronization, could be potential contributors to modulation of MU rate coding in response to both mechanical (Germer et al., 2020) and electrical (Kouzaki et al., 2012) stimuli. Future studies involving the analysis of PCI and MU synchronization are warranted to improve the understanding of neural mechanisms underpinning NMES.

Nevertheless, contrary to our hypotheses, no differences in MU DR were found between ISO, NMES and NMES + ISO at any force level although all three conditions led to higher MU DR compared to baseline, at least at moderate force levels. An increase in DR following voluntary exercise was previously reported, although some authors indicated higher DR during submaximal contractions (Patten and Kamen, 2000; Patten et al., 2001; Vila-Chã et al., 2010; Del Vecchio et al., 2019), whereas others showed marked increases in MU DR only during maximal target force (Kamen and Knight, 2004). Conversely, a recent study that analyzed MU discharge patterns following an acute intervention based on whole-body vibration, indicated no differences in MU DR at any force level after the vibration conditions (Lecce et al., 2023), suggesting that exposure to NMES might elicit changes in the MU behavior which differ from those induced by vibration-based exercise.

The present study also showed a significant reduction of MU RT at 50% target force in the ISO condition. These findings indicate that MUs are recruited earlier during the ascending phase of the trapezoidal trajectory performed with a moderate force target where, normally, a greater number of fast and slow twitch muscle fibers are involved during the contraction. A reduction in MU RT during submaximal tasks was also found in previous studies involving strength training (Patten and Kamen, 2000; Del Vecchio et al., 2019). With regards to acute interventions, Lecce et al. (2023) indicated no changes in MU RT after a whole-body vibration exercise, whereas Suzuki et al. (Suzuki et al., 1990) reported a decrease in the MU RT following successive, moderate force isometric contractions. The authors justified such modulation with an altered excitability of the agonist motoneuron pool (Suzuki et al., 1990). Del Vecchio et al. (2018) argued that changes in recruitment threshold could be associated with the neuromechanical delay (delay between neural drive and force development during tasks) which could be influenced by muscle fiber composition and tendon stiffness. The authors indicated that training at high force could lead to reduction in musculotendinous stiffness which is strictly related to neuromechanical delay (Grosset et al., 2009). Nevertheless, the acute exercise conditions that were used in the present study unlikely promoted any changes in the muscle-tendon structures (i.e., decrease in musculotendinous stiffness) and this might explain the absence of any major difference in MU RT and DERT. Furthermore, as we reported no significant differences in MU DR at recruitment and derecruitment, this also indicates that there may have been no changes in the control of force during the ramp-up and ramp-down phases of the trapezoidal isometric contractions after the experimental conditions.

In contrast with to our hypothesis, we found no differences in the input–output gain of the tibialis anterior motoneuron pool, across conditions and between pre/post-exercise assessments. This parameter represents the relationship between the change in DR and RT from recruitment to target force and provides an indirect measure of net the excitatory input to the spinal motoneurons. The findings of the present study seem to indicate that acute exercises, including NMES+, did not promote any changes in the motoneuron excitability. However, this relationship is only an indirect estimation of the excitatory input, therefore we cannot ultimately exclude changes at the motoneuron level. Indeed, recent studies demonstrated that spinal H-reflexes were strongly potentiated following NMES superimposed on voluntary contractions while a depression of the spinal reflex response was observed after passive NMES (Lagerquist et al., 2012; Borzuola et al., 2020). These findings indicate acute adaptations in motoneuron excitability which could be related to presynaptic (e.g., presynaptic inhibition of Ia afferent pathways) or supraspinal mechanisms. Understanding how changes in cortical activation affect the modulation of neural drive in response to NMES interventions requires an additional effort as findings from previous studies have been inconclusive and, often, did not provide any information about volitional muscle activity. The use of high-resolution electroencephalography (HR-EEG) could be key to evaluate cortical activation underlying muscle activity during volitional assessments as those commonly performed during HDsEMG measurements. Some authors suggested that performing HDsEMG recordings in combination with HR-EEG would allow a valid estimation of the coherence between cortical and motoneuronal signals in the frequency domains (Gallego et al., 2015; Holobar et al., 2018).

In conclusion, the present work indicated that NMES superimposed on voluntary isometric ankle dorsi flexions promoted acute adaptations at the motor unit level in the tibialis anterior muscle although only at moderate and high force targets. These findings support the evidence that intervention based on the application of NMES superimposed on voluntary exercise might carry specific adjustments at the motor unit level. However the results of this study did not indicate a substantial difference in discharge characteristics between the three experimental conditions. Future studies are warranted to elucidate the corticospinal mechanisms related to the adjustments occurring at the motor unit level. In addition, longitudinal studies are warranted to investigate the modulation of MUs discharge patterns in response to chronic exposure to NMES+.

## Data Availability

The raw data supporting the conclusion of this article will be made available by the authors, without undue reservation.
